# Dipeptidyl Peptidase IV Inhibitor MK-0626 Attenuates Pancreatic Islet Injury in Tacrolimus-Induced Diabetic Rats

**DOI:** 10.1371/journal.pone.0100798

**Published:** 2014-06-24

**Authors:** Long Jin, Sun Woo Lim, Kyoung Chan Doh, Shang Guo Piao, Jian Jin, Seong Beom Heo, Byung Ha Chung, Chul Woo Yang

**Affiliations:** 1 Convergent Research Consortium for Immunologic Disease, Seoul St. Mary’s Hospital, The Catholic University of Korea, Seoul, Korea; 2 Transplant Research Center, The Catholic University of Korea, Seoul, Korea; 3 Division of Nephrology, Department of Internal Medicine, Seoul St. Mary’s Hospital, The Catholic University of Korea, Seoul, Korea; Université Paris Descartes, France

## Abstract

**Background:**

Tacrolimus (TAC)-induced pancreatic islet injury is one of the important causes of new-onset diabetes in transplant recipients. This study was performed to evaluate whether a dipeptidyl peptidase IV (DPP IV) inhibitor is effective in improving TAC-induced diabetes mellitus by reducing pancreatic islet injury.

**Methods:**

Rats were treated with TAC (1.5 mg/kg, subcutaneously) and the DPP IV inhibitor MK-0626 (10 or 20 mg/kg, oral gavage) for 4 weeks. The effect of MK-0626 on TAC-induced diabetes was evaluated by assessing pancreatic islet function, histopathology. TAC-induced incretin dysfunction was also examined based on active glucagon-like peptide-1 (GLP-1) levels in the serum after glucose loading. The protective effect of MK-0626 was evaluated by measuring markers of oxidative stress, oxidative resistance, and apoptosis. To determine whether enhanced GLP-1 signaling is associated with these protective effects, we measured the expression of the GLP-1 receptor (GLP-1R) and the effect of the GLP-1 analog exendin-4 on cell viability and oxidative stress in isolated islets.

**Results:**

MK-0626 treatment attenuated TAC-induced pancreatic islet dysfunction and islet morphology. TAC treatment led to a defect in active GLP-1 secretion; however, MK-0626 reversed these effects. TAC treatment increased the level of 8-hydroxy-2′-deoxyguanosine (8-OHdG), the number of apoptotic death, and the level of active caspase-3, and decreased the level of manganese superoxide dismutase and heme oxygenase-1; MK-0626 treatment reversed these changes. MK-0626 treatment restored the expression of GLP-1R, and direct administration of exendin-4 to isolated islets reduced TAC-induced cell death and 8-OHdG expression.

**Conclusions:**

The DPP IV inhibitor MK-0626wasan effective antidiabetic agent that exerted antioxidative and antiapoptotic effects via enhanced GLP-1 signaling in TAC-induced diabetics.

## Introduction

Tacrolimus (TAC) is a widely used maintenance immunosuppressant in renal transplant recipients (KTR). However, it causes considerable metabolic derangement. In particular, new-onset diabetes after transplantation (NODAT), which occurs in 10%–25% of the patients receiving TAC, has emerged as a major adverse event of this drug [1], [2]. This condition leads to serious consequences, including reduced graft survival and increased risk of infectious and cardiovascular diseases. Although the pathogenesis of NODAT caused by TAC remains undetermined, this condition is partly related to the direct toxic effect of TAC on pancreatic β cells, and oxidative stress plays a pivotal role in TAC-induced pancreatic islet dysfunction [3], [4].

Highly selective dipeptidyl peptidase IV (DPP IV) inhibitors are quite different from conventional antidiabetic agents and control hyperglycemia by stimulating insulin production via the prevention of the degradation of two major incretins, the glucagon-like peptide-1 (GLP-1) and the glucose inhibitory peptide (GIP) [5]–[7]. In addition, DPP IV inhibitors have protective effects against inflammation, oxidative injury, and apoptotic cell death in various disease models [8]–[12]. Considering their antidiabetic and tissue-protective effects, the use of DPP IV inhibitors may be ideal in patients with TAC-induced diabetes. However, it remains unclear whether TAC-induced diabetes is associated with incretin dysfunction, and whether the tissue-protective effects of DPP IV inhibitors are also effective in TAC-induced pancreatic islet cell injury.

Therefore, we designed this study to assess the effect of a DPP IV inhibitor on TAC-induced diabetes. First, we evaluated incretin dysfunction in the setting of an animal model of TAC-induced diabetes. Second, we tested whether the DPP IV inhibitor effectively controlled TAC-induced hyperglycemia. Third, we evaluated whether the protective effect of the DPP IV inhibitor was also present in TAC-induced pancreatic islet injury. We expect that the results of our study will provide a rationale for the use of DPP IV inhibitors in patients with NODAT caused by TAC.

## Methods

### Animals and Drugs

The Animal Care and Use Committee of the Catholic University of Korea approved the experimental protocol (CUMC-2012-0117-02), and all procedures performed in this study were in accordance with ethical guidelines for animal studies. Eight-week-old male Sprague Dawley rats (Charles River Technology, Seoul, Korea) that initially weighed 220–230 g were housed in cages (Nalge Co., Rochester, NY) in a controlled-temperature and controlled-light environment at the Catholic University of Korea’s animal care facility. The rats received a low-salt diet (0.05% sodium, Teklad Premier, Madison, WI). Tacrolimus (TAC, Prograft, Astellas Pharma Inc., Ibaraki, Japan) was diluted in olive oil (Sigma, St. Louis, MO) to a final concentration of 1 mg/mL. DPP IV inhibitor MK-0626 was kindly supplied by Merk Sharp & Dohme Corp (Kenilworth, NJ), and was diluted in drinking water to a final concentration of 10 or 20 mg/mL.

### Experimental Design

The first study was designed to determine the dose with a relevant therapeutic level in rats. We administered three different doses of MK-0626 (10, 20, and 40 mg/kg, oral gavage) and TAC (1.5 mg/kg, s.c.) to rats for 4 weeks and chose the optimal doses of MK-0626 to be used in the second study. Based on the first study results, the second study was designed to evaluate the effect of MK-0626 on TAC-induced pancreatic islet injury. After acclimatization and a low-salt diet for 1 week, weight-matched rats were randomized to six groups containing eight rats each and were treated daily with TAC (1.5 mg/kg) or control (olive oil, 1 mg/mL) with or without MK-0626 (M, 10 and 20 mg/kg) for 4 weeks. Routes of administrating drugs were chosen based on the first study.

### Basic Protocol

After 1 week of a low-salt diet, weight-matched rats were randomly assigned to the different treatment groups. Rats were pair-fed and their body weight was monitored daily. TAC-induced diabetes was defined by two-hour plasma glucose around 200 mg/dL or higher during IPGTT on consecutive measurements according to the guideline from the American diabetes association. After the 4-week treatments, animals were anesthetized with Zoletil 50 (10 mg/kg, intraperitoneally; Virbac Laboratories) and Rompun (15 mg/kg, intraperitoneally; Bayer, Leuverkusen, Germany), and blood samples and tissue specimens were obtained for further analysis. Whole-blood TAC level was measured according to methods described previously [13], [14].

### Preservation of Pancreatic Tissues

Pancreases were preserved by in vivo perfusion through the abdominal aorta. The animals were perfused with 0.01 mol/L phosphate-buffered saline to flush blood from the tissues. Dissected pancreases were immersed in periodate-lysine-2% paraformaldehyde solution and embedded in paraffin for further histologic observation.

### Pancreatic Beta Cell Function

An intraperitoneal glucose tolerance test (IPGTT) was performed at the end of the 4 week treatment period as previously described [13], [14], and the area under the curve of glucose (AUCg) was calculated by trapezoidal estimation from the values obtained in the IPGTT. The plasma insulin level was measured in duplicate by an enzyme-linked immunosorbent assay kit (Dainabot Corporation, Tokyo, Japan). HbA1c levels were measured using the hemoCue B-Glucose Analyzer (HemoCue AB, Angelholm, Sweden) and the DCA 2000+HbA1c kit (Bayer, Elkhart, IN). The HOMA-R index was calculated using the following formula: HOMA-IR = fasting insulin (international units/mL)×fasting glucose (mmol/L)/22.5.

### Measurement of Pancreatic Beta Cell Area

A minimum of 20 filed per section were assessed using a color image analyzer (TDI Scope EyeTM Version 3.0 for Window; Olympus, Tokyo, Japan). Briefly, captured images from immunohistochemistry with insulin were quantified using the Polygon program by measuring the pancreas area seen to contain insulin-positive area except vacuoles when viewed under x200 magnifications. Histopathologic analysis was performed on randomly selected fields of pancreas section by a pathologist blinded to the identity of the treatment groups.

### Measurement of the Levels of DPP IV Activity and GLP-1

After 4 weeks of treatment, the animals were fasted for approximately 24 h. Blood samples were drawn from tail veins at 0, 3.75, 7.5, 15, 30, and 60 min after glucose loading (2 g/kg). To determine the activity of GLP-1 (uncleaved, 7–36 amide or 7–37) in the serum, blood samples were collected into tubes containing a DPP IV inhibitor (50 µM), centrifuged, and frozen at –80°C. To measure plasma DPP-IV activity, blood samples were collected into prechilled, EDTA-treated tubes, centrifuged at 4°C, separated, and frozen at –80°C. The level of active GLP-1 (Millipore Corporation, Billerica, MA) and DPP IV activity (Merck KGaA, Darmstadt, Germany) were measured using a commercially available ELISA kit, according to the manufacturer’s recommendations.

### Measurement of 8-Hydroxy-2′-Deoxyguanosine

Oxidative DNA damage was evaluated based on the level of the DNA adduct 8-hydroxy-2*′*-deoxyguanosine (8-OHdG) in serum, 24 h urine, and culture media using a competitive enzyme-linked immunosorbent assay (Cell Biolabs, San Diego, CA).

### Immunohistochemistry

Immunohistochemistry was performed as described previously [13], [15]. The GLP-1 receptor (GLP-1R), insulin, and 8-OHdG were detected in 4 µm tissue sections by incubating cells for 12 h with specific antibodies against GLP-1R (Abcam, Cambridge, MA), insulin (Zymed, San Francisco, CA), 8-OHdG (JaICA, Shizuoka, Japan) at 4°C.

### Immunoblot Analysis

Immunoblot analysis was performed as described previously [13], [15]. Using tissue lysates from the isolated pancreatic islets of experimental animals, GLP-1R, active caspase-3, heme oxygenase-1 (HO-1), HO-2, and β-actin were detected by incubating for 12 h with specific antibodies against GLP-1R (Abcam), active caspase-3 (Millipore), MnSOD (Abcam), HO-1 (Enzo Life Sciences, Farmingdale, NY), HO-2 (Enzo Life Science), and β-actin (Sigma) at 4°C.

### In situ TdT-mediated dUTP–biotin Nick End Labeling Assay

Apoptosis in tissue sections was identified using the ApopTag in situ apoptosis detection kit (Millipore). The number of TUNEL-positive cells was counted in 20 different fields in each section at 200x magnification.

### Islet Isolation and Cell Culture

Pancreatic islets were isolated from normal male Sprague Dawley rats (250–300 g) by collagenase digestion and separated by discontinuous gradient purification, as described previously [16], [17]. Isolated islets were treated with TAC (60 ng/mL) and/or exendin-4 (Exd) (Sigma; 10 or 50 µM) or N-acetylcysteine (NAC) (Sigma; 50 or 100 µM) for 24 h and were stained with 0.67 mmol/L acridine orange (AO) and 75 mmol/L propidium iodide (PI) (Sigma). The viability of islet cells was referred to the previous reports [17]–[19]. The experiments were conducted at least three times. The experiments were performed with individual samples from separate experiments and not using different wells from the same culture plate.

### Statistical Analysis

The data are expressed as means ± standard error of at least three independent experiments. Multiple comparisons between groups were performed by one-way ANOVA with the Bonferroni post hoc test (SPSS software version 19.0; IBM, Armonk, NY). Statistical significance was assumed as P<0.05.

## Results

### Determination of the Therapeutic Doses of MK-0626

To choose relevant therapeutic doses of MK-0626 in rats, we tested three different doses of MK-0626 in tacrolimus (TAC)-treated rats. Table 1 shows the results of intraperitoneal glucose tolerance test (IPGTT) and levels of TAC. The IPGTT showed that 10 and 20 mg/kg of MK-0626 treatment yielded a lower blood glucose level from 60 to 120 min after glucose loading compared with the TAC group. However, 40 mg/kg of MK-0626 treatment yielded a higher blood glucose level and TAC trough level than the TAC group. Based on these findings, we chose 10 and 20 mg/kg of MK-0626 for the combination study.

**Table 1:** Determination of the therapeutic doses of MK-0626 on IPGTT and trough level of tacrolimus in rats.

	Blood glucose level (mg/dL)a					TAC level (ng/mL)
	0 min	30 min	60 min	90 min	120 min	
Control (*n* = 8)	87±2	291±25	174±8	131±4	115±2	-
M10 (*n* = 8)	94±3	253±13	153±3^a^	121±4	113±3	-
M20 (*n* = 8)	95±2	252±23	152±8	109±3	106±3	-
M40 (*n* = 8)	95±2	232±22	146±6^a^	120±3	109±2	-
TAC (*n* = 8)	93±7	467±14^a^	312±17^a^	217±13^a^	185±5^a^	6.7±0.5
TAC+M10 (*n* = 8)	105±3	405±31	258±13^b^	191±13^b^	150±10^b^	6.8±0.4
TAC+M20 (*n* = 8)	96±5	389±35	226±23^b^	176±14^b^	139±7^b^	7.9±0.4
TAC+M40 (*n* = 8)	96±4	514±9^b^	303±20	216±16	178±14	8.5±0.5^b^

a Indicates the results of intraperitoneal glucose tolerance test. Values are means ± SE. n, No. of animals. M10, M20, and M40, 10, 20 and 40 mg/kg of MK-0626; TAC, tacrolimus; IPGTT, intraperitoneal glucose tolerance test; TAC level, trough blood level of TAC.

a P<0.05 vs. control;

b P<0.05 vs. TAC.

### MK-0626 Treatment Attenuates TAC-induced Pancreatic Islet Dysfunction and Reduction of Islet Size

**Figure 1:**
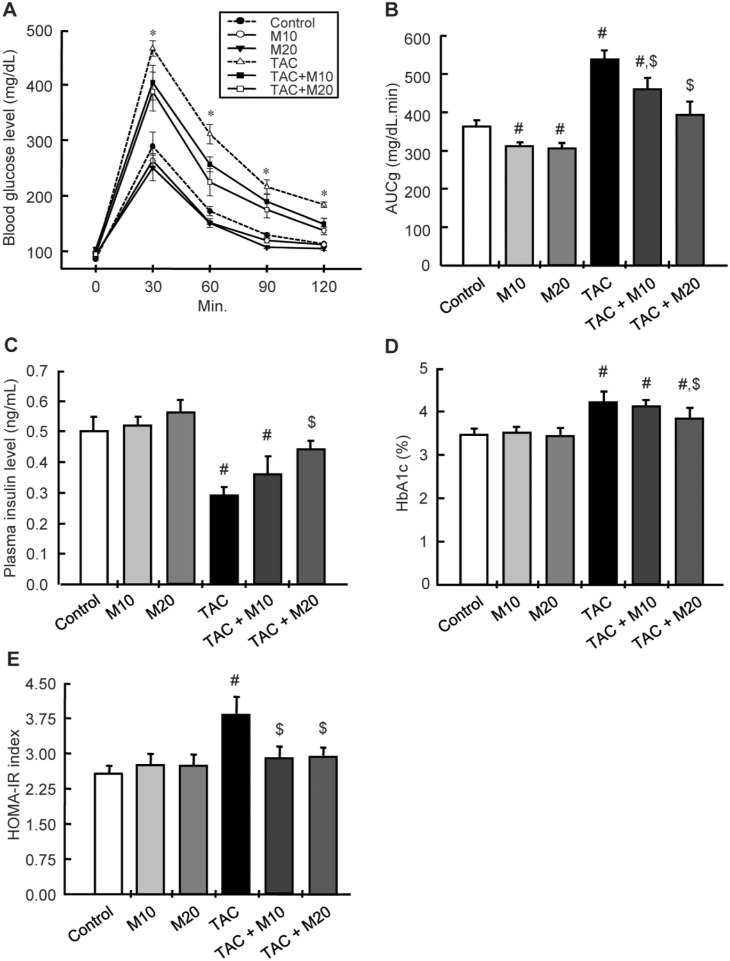
Effect of MK-0626 on TAC-induced pancreatic islet dysfunction. (A) Intraperitoneal glucose tolerance test (IPGTT). (B) The area under the curve for glucose (AUCg). (C) Plasma insulin level. (D) Hemoglobin A1c (HbA1c). (E) Homeostatic model assessment of insulin resistance (HOMA-IR) index. Note that all parameters are recovered after combined treatment with MK-0626 (M) and tacrolimus (TAC). *n* = 8 rats per group. ^#^P<0.05 vs. Control. ^$^P<0.05 vs. TAC, *P<0.05 vs. other groups.

**Figure 2:**
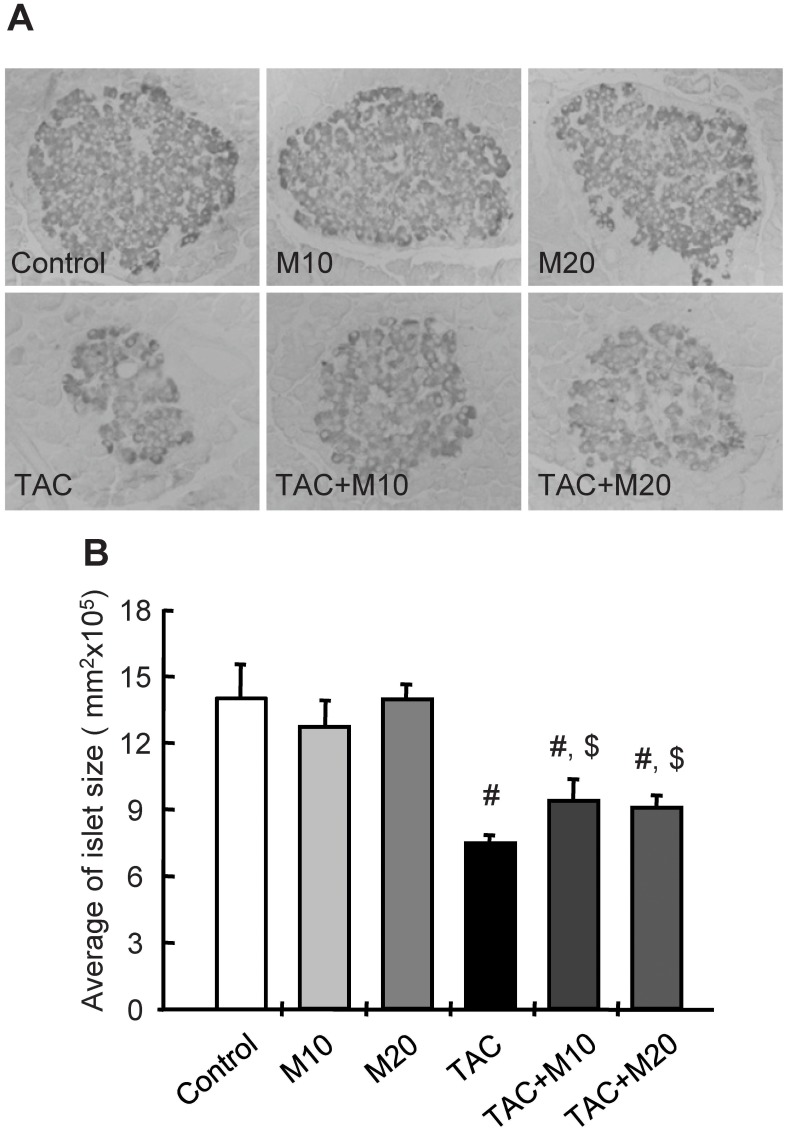
Effect of MK-0626 on pancreatic islet morphology and size during tacrolimus-induced injury. Immunohistochemistry for insulin showing pancreatic islet morphology and size in the experimental groups. Well-preserved pancreatic islet structure and insulin-immunoreactivity areas are seen in the control alone and MK-0626 (M) groups. The tacrolimus (TAC) group showed a smaller size of islet with lower intensity of insulin staining within islet than control group. In contrast, additional treatment of MK-0626 reversed these changes (A). Quantitative analysis of islet area. Note the significant lower islet area in the TAC+M10 and TAC+M20 groups than in the TAC group (B). Original magnifications, x 400. *n* = 8 rats per group, ^#^P<0.05 vs. control. ^$^P<0.05 vs. TAC.

Four weeks of TAC treatment increased blood glucose levels, hemoglobin A1c (HbA1c), the homeostatic model assessment of insulin resistance (HOMA-IR) index, and decreased insulin level. MK-0626 treatment was reverted these changes, as it decreased blood glucose level, HbA1c, and HOMA-IR, and increased insulin level compared with the TAC group (Figure 1). The TAC group showed a smaller islet with lower intensity of insulin staining within islet, and this effect was reverted by MK-0626 treatment (Figure 2).

### MK-0626 Treatment Recovers TAC-induced GLP-1 Deficiency

**Figure 3:**
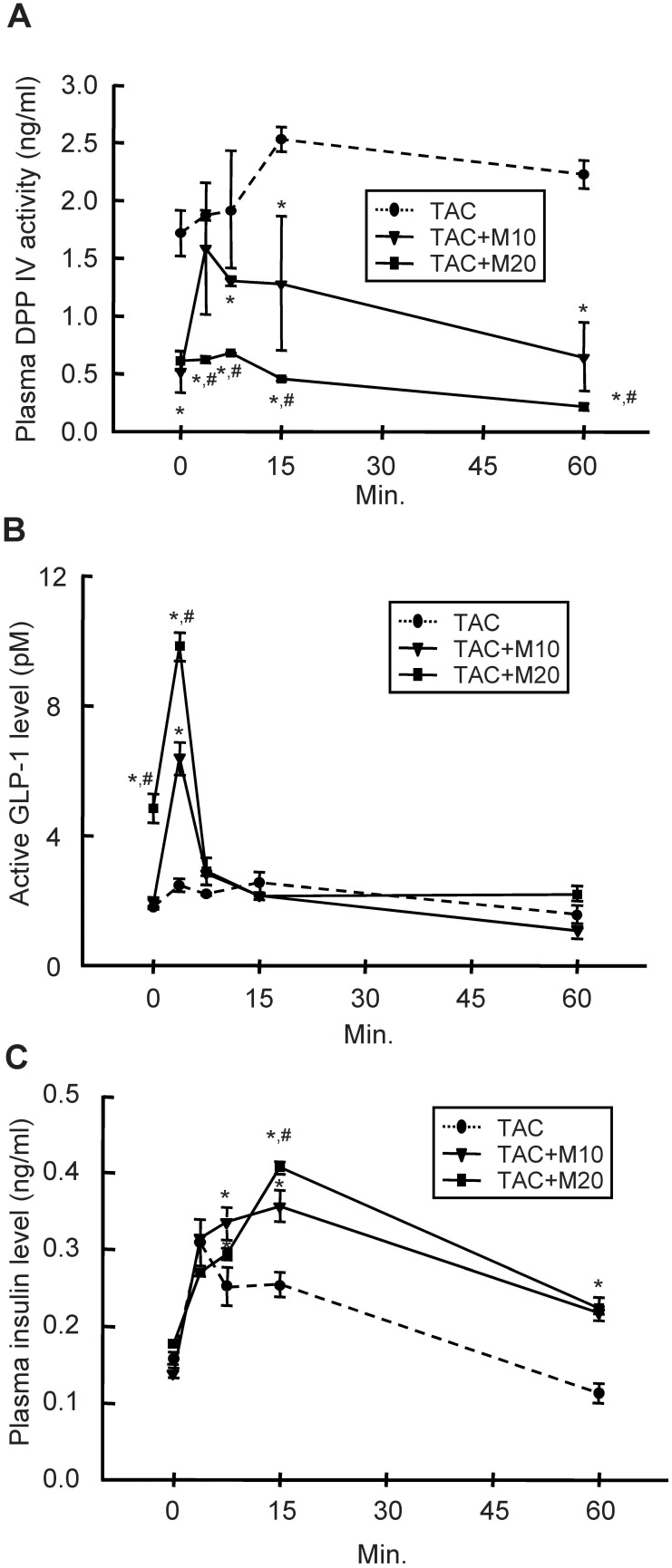
Changes in the level of DPP IV, GLP-1, and insulin after glucose loading in experimental rats. Tacrolimus (TAC)-and/or MK-0626 (M)-treated rats were given glucose at 0 min, followed by the measurement of plasma DPP IV activity, serum active GLP-1 level, and plasma insulin level at 0, 3.75, 7.5, 15, and 60 min. (A) MK-0626 treatment significantly inhibits DPP IV activity, and GLP-1 level reach a higher level in MK-0626 combined groups compared with the TAC group (B). (C) Note that plasma insulin level is sustained until 60 min in TAC plus MK-0626-treated groups compared with the TAC group. *n* = 8 rats per group. *P<0.05 vs. TAC. ^#^P<0.05 vs. TAC+M10.

After a 4-week treatment with TAC, plasma DPP IV activity was significantly increased (1.7±0.2 ng/ml in the TAC group vs. 0.9±0.1 ng/mL in the control group, P<0.05), whereas serum active GLP-1 levels were lower (1.8±0.01 pM in the TAC group vs. 8.7±0.5 pM in the control group, P<0.05) than that observed in the control group. Based on these results, we evaluated the effect of MK-0626 on plasma DPP IV activity, serum active GLP-1 response, and plasma insulin excursions in glucose-loaded rats after 24 h fasting (Figure 3). Combined treatment of TAC and MK-0626 significantly decreased plasma DPP IV activity compared with the TAC group, in a dose-dependent manner. Concomitantly, the high level of active GLP-1 was responded in the TAC plus MK-0626 groups within ten minutes after glucose loading, whereas the TAC group showed blunt GLP-1 excursion. Furthermore, the profound elevation of the insulin profile was observed in the TAC plus MK-0626 groups compared with the TAC group.

### MK-0626 Attenuates TAC-induced Oxidative Injury in Pancreatic Islet

**Figure 4:**
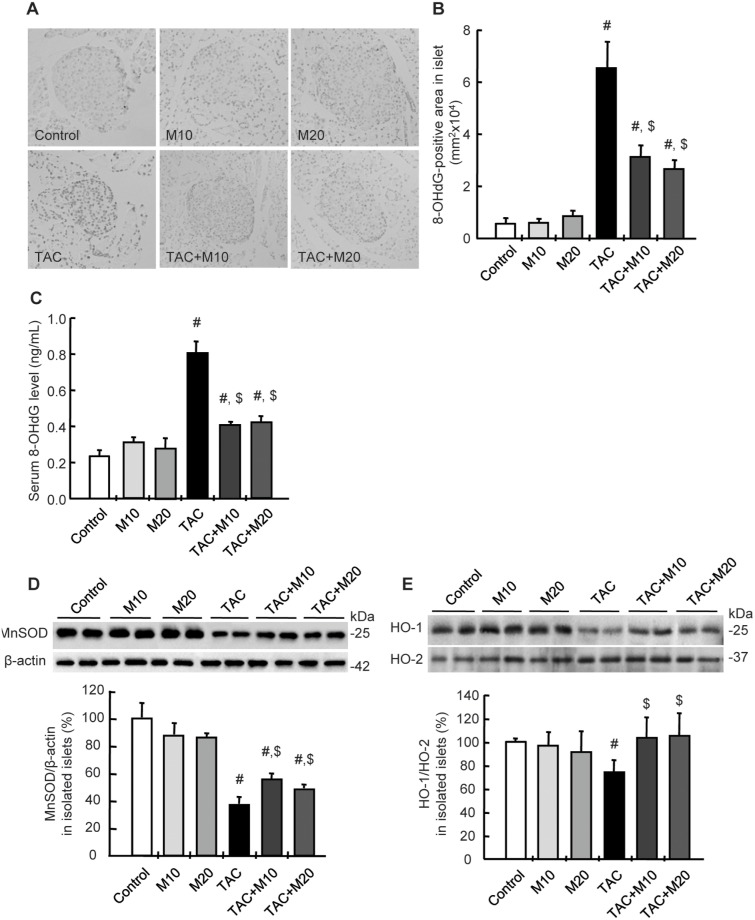
Effect of MK-0626 on the expression of 8-hydroxy-2′-deoxyguanosine, manganese superoxide dismutase and heme oxygenase-1 during tacrolimus-induced pancreatic islet injury. (A and B) Immunohistochemistry for 8-hydroxy-2′-deoxyguanosine (8-OHdG) and its quantitative analysis in islet show intense nuclear expression and larger positive area for 8-OHdG in tacrolimus (TAC) groups. These changes are markedly decreased in TAC plus MK-0626 (M)-treated groups. (C) The TAC-induced serum 8-OHdG level is lowered by the addition of MK-0626. Immunoblot analysis of manganese superoxide dismutase (MnSOD) (D) and heme oxygenase-1 (HO-1, inducible form) and HO-2 (constitutive form) (E) in isolated pancreatic islets from the experimental rats. Note that the level of MnSOD and HO-1/HO-2 is lower in the TAC group; however, MK-0626 treatment leads to the recovery of its expression. Original magnifications, x400. *n* = 8 rats per group. ^#^P<0.05 vs. control. ^$^P<0.05 vs. TAC.

8-hydroxy-2′-deoxyguanosine (8-OHdG) was used as a marker of oxidative DNA damage, Figure 4 shows the results of immunohistochemistry for 8-OHdG and the levels of serum 8-OHdG in the experimental groups. The strong nuclear expression of 8-OHdG in islets and its area of positive staining were markedly increased in the TAC group compared with the control group; this effect was reversed after MK-0626 treatment. A higher level of 8-OHdG in the serum was also detected in the TAC group; MK-0626 treatment significantly decreased these changes. We further evaluated whether MK-0626 affects the expression of anti-oxidative stress related molecules, manganese superoxide dismutase (MnSOD), heme oxygenase-1 (HO-1, inducible form) and HO-2 (constitutive form), and expressed it as the ratio of HO-1/HO-2. Their expression in isolated islets from experimental rats was reduced in the TAC group compared with the control group, and was recovered by MK-0626 treatment.

### Mk-0626 Treatment Attenuates Tac-induced Apoptotic Cell Death

**Figure 5:**
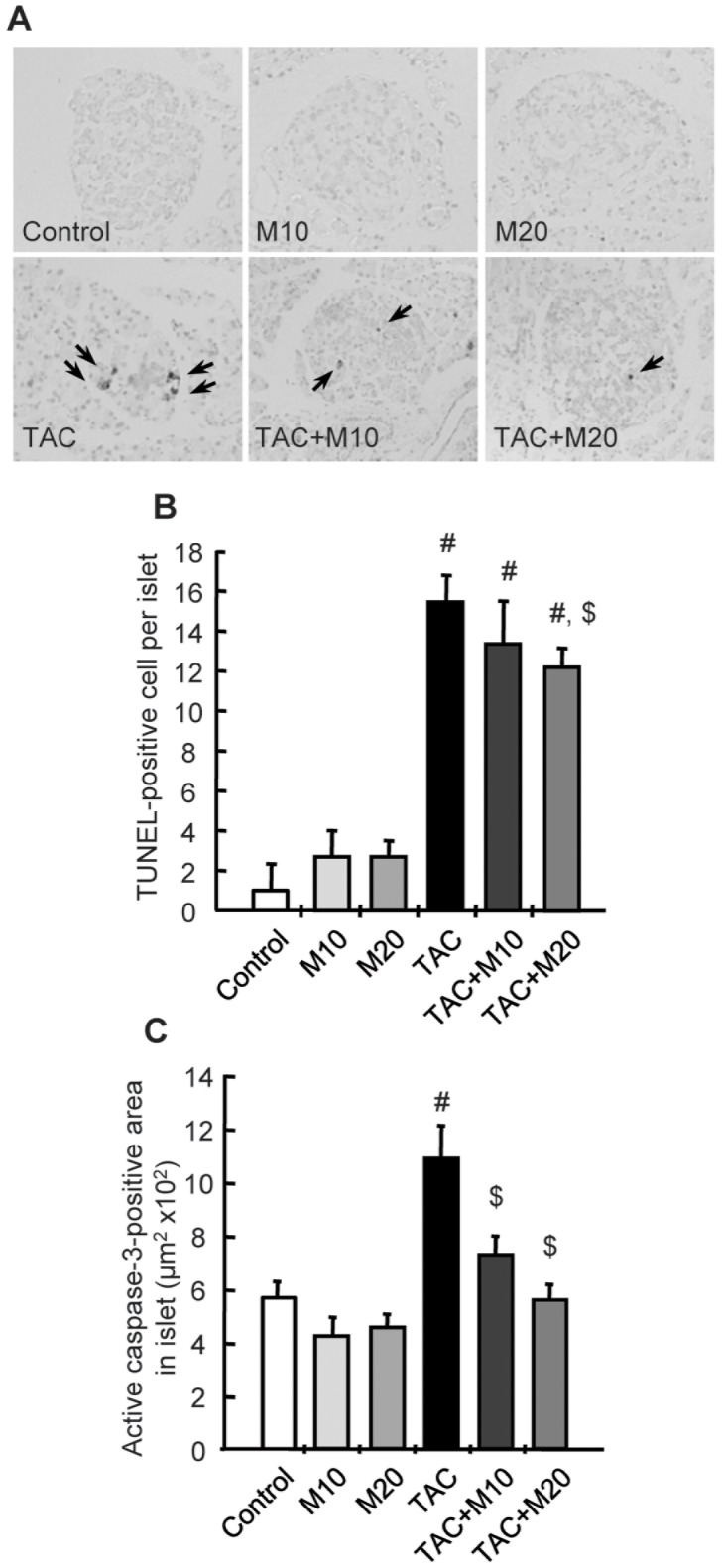
Effect of MK-0626 on apoptosis during tacrolimus-induced pancreatic islet injury. In situ TdT-mediated dUTP–biotin nick end labeling (TUNEL) assay (A) and its analysis (B) to detect apoptosis in pancreatic islets in the experimental groups. MK-0626 (M) treatment significantly reduces TUNEL-positive cells (arrows) compared with tacrolimus (TAC) group. (C) Quantitative analysis in islets of the active form of caspase-3 in the experimental groups. Note that the MK-0626 treatment significantly reduced active caspase-3 compared with the TAC group. Original magnifications, x400. *n* = 8 rats per group. ^#^P<0.05 vs. control. ^$^P<0.05 vs. TAC.

Next, we evaluated whether MK-0626 treatment would affects apoptotic cell death, an important mechanism of cell death in TAC-induced pancreatic injury. Apoptosis is identified by condensation and fragmentation of the nucleus and shrinkage of the cells in light micrographs using TUNEL assay method. The number of TUNEL-positive cells within the islets was significantly higher in the TAC group compared with the control group, and this was reduced by addition of MK-0626. The number of the active form of caspase-3 in the islets was also decreased by addition of MK-0626 compared with those observed in the TAC group (Figure 5).

### MK-0626 Treatment Restores the Pancreatic Islet GLP-1 Receptor Expression in TAC-induced Injury

**Figure 6:**
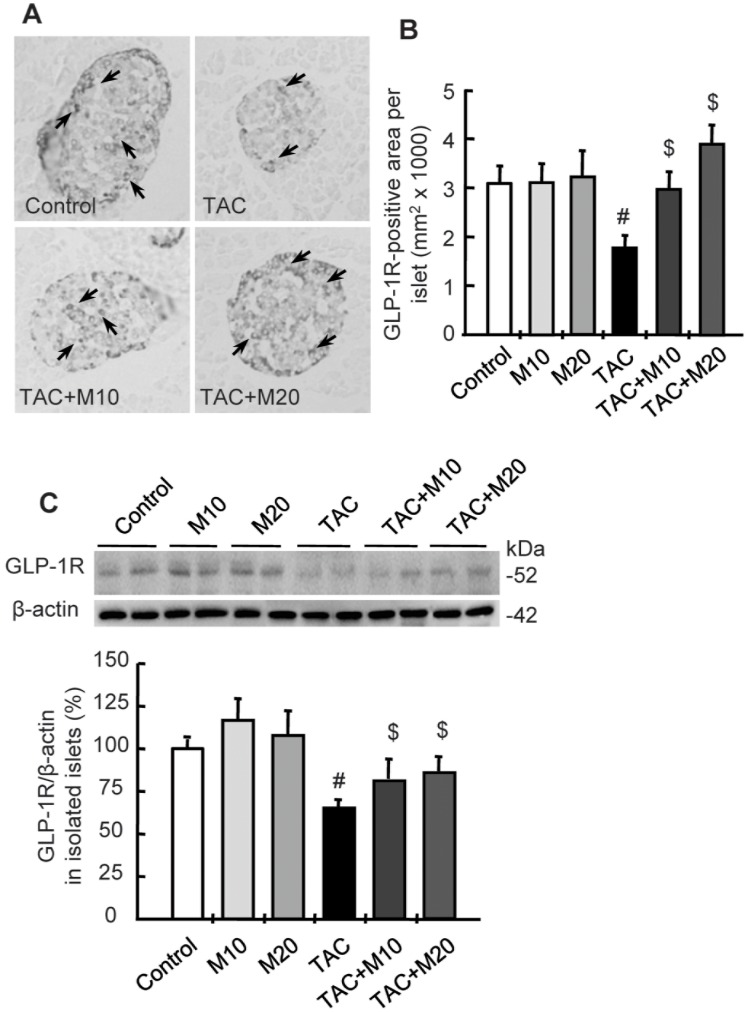
Effect of MK-0626 on the expression of GLP-1 receptor during tacrolimus-induced pancreatic islet injury. Immunohistochemistry (A) and quantitative analysis (B) of GLP-1 receptor (GLP-1R) in islets from tissue sections. Arrows indicate GLP-1R positive cells. (C) Immunoblot analysis of GLP-1R in isolated pancreatic islet from experimental groups. Note that the number and level of GLP-1R is lower in the tacrolimus (TAC) group, but MK-0626 (M)-treated groups exhibit a recovery of its expression compared with the TAC group. Original magnifications, x1000. *n* = 8 rats per group. ^#^P<0.05 vs. control. ^$^P<0.05 vs. TAC.

Protective properties e.g. anti-oxidative stress and anti-apoptosis of DPP IV inhibitor are associated with the activation of GLP-1 and its receptor [9], [12], [20]. Therefore, we performed immunohistochemistry and immunoblot-based quantitative analysis of GLP-1 receptor (GLP-1R) in islets tissues. GLP-1R in islets showed increased immunoreactivity of GLP-1R by addition of MK-0626 compared with the TAC group. Consistently, the protein amount of GLP-1R in isolated pancreatic islets from experimental rats was also lower in the TAC group, and addition of MK-0626 restored these levels, as shown in Figure 6.

### GLP-1 Analog Exendin-4 Treatment Attenuates TAC-Induced Cell Death and Oxidative Stress In vitro

**Figure 7:**
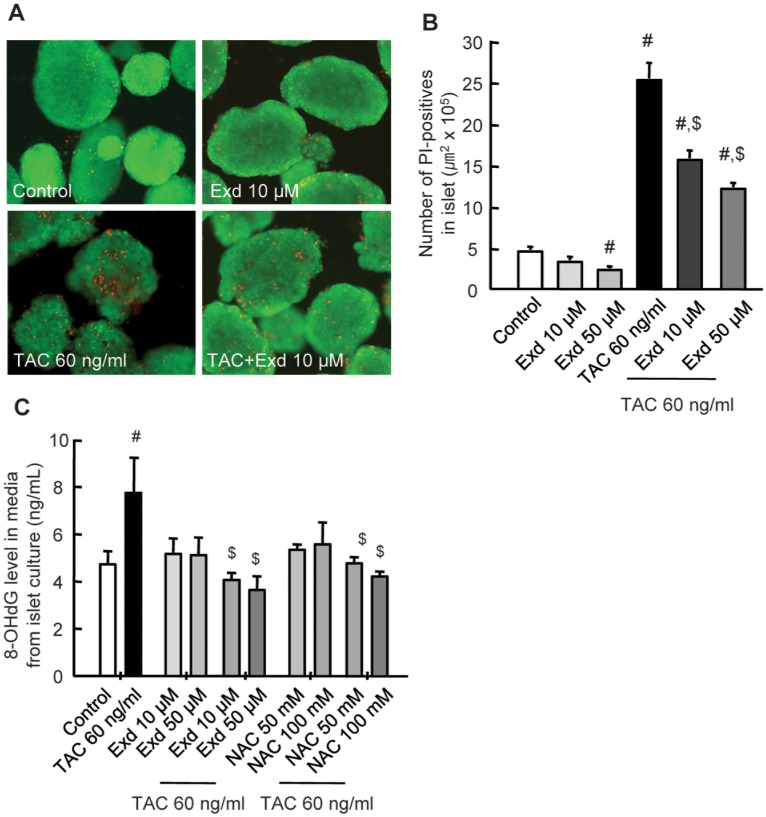
Effect of GLP-1 analog exendin-4 on tacrolimus-induced cell death and oxidative stress in vitro. In vitro assay using primary rat islets after treatment with tacrolimus (TAC) and/or exdendin-4 (Exd) or *N*-acetylcysteine (NAC) for 24 h. (A) Acridine orange/propidium iodide (AO/PI) staining to assess the viability of islets. (B) The number of PI-stained cells (red fluorescence) per islet. (C) The 8-OHdG level in culture media from the same experiments of islets cells. PI-positive cell death and 8-OHdG level are significantly reduced in Exd-treated groups compared with the TAC-alone-treated group. These results are confirmed using NAC, which is an antioxidant drug, in the same experiments. Original magnifications, x10. ^#^P<0.05 vs. control. ^$^P<0.05 vs. TAC.

To reveal the exact role of GLP-1, we directly treated GLP-1 analog exendin-4 (Exd) to the primary rat islet with TAC in a culture setting. Acridine orange/propidium iodine (AO/PI) staining to assessed the viability of islet reveals that control islets and Exd-treated islets exhibited an almost uniform green fluorescence, without red fluorescence, which implies the presence of intact plasma membranes were preserved well. However, islets that were exposed to TAC had weak green fluorescence (AO) with number of red fluoresce (PI) within islets; these effects were significantly reversed in the presence of combination treatment with Exd (Figure 7A and B). To evaluate the influence of Exd on TAC-induced oxidative stress, we measured 8-OHdG level in culture media from the experiments described above. The increased 8-OHdG level in the presence of TAC was significantly reduced in the MK-0626- or N-acetylcysteine (NAC as an antioxidant control)-treated group compared with TAC treatment alone (Figure 7C).

## Discussion

The results of our study demonstrated clearly that the DPP IV inhibitor MK-0626hasa protective effect against TAC-induced pancreas islet injury. In the in vivo study, MK-0626 treatment improved the TAC-induced diabetes mellitus by recovering the DPP IV/GLP-1 system. Moreover, it attenuated pancreatic islet dysfunction with histologic improvement in TAC-treated rats. In the in vitro study, direct administration of GLP-1 to the islet decreased oxidative stress and cell death. These findings suggest that the DPP IV inhibitor not only exerts glycemia-lowering effects but also affords tissue protection in TAC-induced diabetic mellitus.

First, we performed a preliminary study to determine the optimal dose of MK-0626, because there is no information on the therapeutic doses of MK-0626 in the setting of TAC-induced pancreatic islet dysfunction in rats. We found that 10 and 20 mg/kg of MK-0626 were effective in controlling hyperglycemia without a significant increase in TAC blood levels; however, higher doses of MK-0626 (40 mg/kg) significantly increased the TAC trough level and exacerbated glucose intolerance, suggesting the presence of a significant drug interaction between MK-0626 and TAC. It is well known that MK-0626 and TAC are metabolized by cytochrome P450 (CYP3A) and that MK-0626 is a substrate of the P-glycoprotein [21], [22]. Therefore, careful monitoring of TAC blood levels should be considered before using high-dose DPP IV inhibitors in transplant recipients, considering the narrow therapeutic range of the two drugs and drug–drug interactions.

Next, we evaluated the rationale for the use of a DPP IV inhibitor in TAC-induced diabetes mellitus. In the present study, chronic TAC treatment caused incretin dysfunction, as demonstrated by increased circulating DPP IV activity and reduced circulating levels of GLP-1and of its receptor in pancreatic islets. Combined treatment with MK-reversed DPP IV/GLP-1 dysregulation and had a significant positive effect on both GLP-1 excursion and insulin secretion while maintaining low DPP IV activity after glucose loading in TAC-treated rats. Consequently, the levels of AUCg, HbA1c, HOMA-IR, and insulin were restored by cotreatment with MK-0626, as shown in Figure 1. These findings suggest that the DPP IV/GLP-1 system is involved in TAC-induced diabetes mellitus and that the preservation of endogenously secreted GLP-1 by treatment with a DPP IV inhibitor is a reasonable approach in the treatment of TAC-induced diabetes.

In addition to controlling hyperglycemia, cotreatment with MK-0626 recovered pancreatic islet size and the immunoreactivity of insulin in islets compared with TAC treatment. To demonstrate its protective effect, we evaluated whether MK-0626 affected TAC-induced oxidative stress, which is a common pathway of TAC-induced toxicity [3], [23]. Our results showed that MK-0626 not only dramatically decreased the level of 8-OHdG, which is a marker of oxidative DNA damage, in islets and in the serum, but also recovered MnSOD and HO-1 expression. In fact, there is increasing evidence of an association between the development of diabetes and increased oxidative stress, and of the function of DPP IV-inhibitor treatment as a scavenger of reactive oxygen species, with a resultant antihyperglycemic effect [13], [24], [25]. Overall, these findings suggest that MK-0626 protects against TAC-induced pancreatic islet injury via a decrease in oxidative stress and subsequent apoptotic cell death.

We evaluated the protective effect of GLP-1 because the DPP IV inhibitor functions to increase incretin levels (e.g., GLP-1 and GIP). Actually, accumulating evidence has showed that the activation of GLP-1R signaling plays an important role in protecting cells (e.g., pancreatic islets and retinal cells) and organs (e.g., kidney and heart) against ischemic reperfusion injury by decreasing oxidative DNA damage and apoptosis [8]–[10], [12], [26], [27]. DPP IV inhibitors exert their tissue-protective effect by enhancing the binding affinity of GLP-1 for GLP-1R [12], [28]. In this study, we found a protective effect of MK-0626 against TAC-induced pancreatic islet injury; however, this effect was not sufficient to explain that this effect is mediated by GLP-1 produced by MK-0626. Therefore, we applied the GLP-1 analog Exd directly to TAC-treated rat islets and found that Exd treatment effectively decreased the oxidative stress and apoptosis caused by TAC, as shown in Figure 7. This finding confirmed the hypothesis that the effect of MK-0626 on TAC-induced pancreatic islet dysfunction is mediated by GLP-1.

The current study revealed that the doses of the DPP IV inhibitor that are necessary for preventing diabetes are also effective in protecting against tissue injury. The observed islet protection may be attributed to a reduction in oxidative stress and subsequent apoptotic cell death, via the activation of the GLP-1/GLP-1R system. Emerging evidence indicates that GLP-1-based therapies may provide benefits for other organ tissues, beyond glycemic control [29], [30]. Thus, we suggest that the use of this DPP IV inhibitor may be useful in the management of diabetes complications, such as diabetic neuropathy and retinopathy, in TAC-induced diabetes mellitus.

The results of our study can be translated into clinical practice. First, the incretin class of agents is suitable as a treatment for NODAT, as shown in our experiments and other clinical studies [31]–[33]. However, a high dose of DPP IV inhibitors may worsen β cell function because of a drug interaction with TAC. Therefore, the dosing of the DPP IV inhibitor is important for avoiding TAC-induced toxicity, and careful monitoring of TAC trough level is required in transplant patients receiving TAC and DPP IV inhibitors. Second, the effect of this DPP IV inhibitor may be extended to other diabetogenic immunosuppressive agents. We reported previously that sirolimus- or tacrolimus-induced pancreatic islet injury is mediated by oxidative stress [3], [34]. Therefore, MK-0626 may be effective against sirolimus-induced pancreatic islet injury, considering its antioxidant and antiapoptotic effects. This presumption may be supported by recent clinical studies that showed that sitagliptin treatment is effective in treating NODAT caused by low-dose tacrolimus and sirolimus [32].

Our study had several limitations. First, we used a low-salt diet in the experimental model of TAC-induced organ injury. A low-salt diet was used to activate the renin–angiotensin system (RAS), because activation of RAS is important in the pathogenesis of TAC-induced renal and pancreatic islet injury [35]–[39]. Second, we were not able to provide a survival curve demonstrating differences in the occurrence of clinically defined diabetes between the treatment groups, as this would have required the frequent administration of the IPGTT, which is stressful to animals.

In conclusion, the DPP IV inhibitor was effective in improving not only hyperglycemia but also TAC-induced pancreatic islet injury. These findings provide a rationale for the use DPP IV inhibitors in transplant patients with NODAT caused by TAC.
